# Combined Effects of Water Depth and Velocity on the Accelerometric Parameters Measured in Horses Exercised on a Water Treadmill

**DOI:** 10.3390/ani10020236

**Published:** 2020-02-03

**Authors:** Aritz Saitua, Mireya Becero, David Argüelles, Cristina Castejón-Riber, Antonia Sánchez de Medina, Katy Satué, Ana Muñoz

**Affiliations:** 1 Equine Sport Medicine Center, School of Veterinary Medicine, University of Córdoba, 14014 Córdoba, Spain; aritz_sp91@hotmail.com (A.S.); mireyabece_1394@hotmail.com (M.B.); 2 Department of Animal Medicine and Surgery, School of Veterinary Medicine, University of Córdoba, 14014 Córdoba, Spain; darguellescap@gmx.es (D.A.); tsmedina189@hotmail.com (A.S.d.M.); 3 Veterinary Teaching Hospital, School of Veterinary Medicine, University of Córdoba, 14014 Córdoba, Spain; 4 Department of Artistic and Body Education, University of Córdoba, 14014 Córdoba, Spain; ccastejon@uco.es; 5 Department of Animal Medicine and Surgery, School of Veterinary Medicine, University Cardenal Herrera-CEU, 46115 Valencia, Spain; ksatue@uch.ceu.es

**Keywords:** exercise, horse, power, rehabilitation, training, water treadmill

## Abstract

**Simple Summary:**

Anecdotal comments of some trainers suggest that exercise on a water treadmill (WT), due to the greater difficulty of movement in water compared to air, increases muscularization and sports performance. Therefore, we studied accelerometric changes in six horses exercised for 40 min on a WT, comparing four situations: without water (DT) and with water at the depth of fetlock (FET), carpus (CAR), at 6 km/h and at the depth of the stifle (STF) at 5 km/h. Another five horses performed the same sessions, but all of them at 5 km/h. We observed that total power (TP) increased from depth DT to FET and CAR, without additional increases at STF depth when the velocity was the same at all depths. However, when the velocity was reduced to 5 km/h, TP decreased at the STF depth. Significant increases in dorsoventral power and dorsoventral displacement (movement of the center of gravity in up-down direction) increased with water depth and velocity. In conclusion, TP during an exercise session on a WT depends on both velocity and water depth, and this power is mainly directed towards the dorsoventral axis. These results should be taken into account when designing a training or rehabilitation plan.

**Abstract:**

Horse trainers often claim that exercise on a water treadmill (WT) leads to a greater muscle power and development compared to terrestrial locomotion, because of the greater viscosity of water compared to air. This research assesses locomotor changes measured with accelerometers fixed in the pectoral region and in the sacrum midline in six horses subjected to exercise sessions of 40 min duration on a WT without water (DT), and with water at the depth of fetlock (FET) and carpus (CAR) with velocities of 6 km/h and at the depth of stifle (STF) at 5 km/h. Another five horses performed the same exercise sessions but always with a velocity of 5 km/h. Total power increased from DT to FET and CAR, without significant differences between CAR and STF depths when the velocity was the same. However, a significant decrease was found when the velocity was reduced. The greater total power with water was distributed mainly to the dorsoventral axis, with significant increases in dorsoventral displacement and dorsoventral power. Both parameters were significantly affected by velocity and water depth. In conclusion, total and dorsoventral powers increased with velocity and water depth, leading to reduction in longitudinal and mediolateral power, during exercise on a WT.

## 1. Introduction

The availability of different systems to exercise horses in the water, such as swimming pools and water treadmills (WTs), has increased in recent years with their increasing popularity, seeing them used in training and in rehabilitation centers worldwide. To the authors’ best knowledge, there is only one published article that describes how, when and why WTs are used for sport horses [1]. Training was the main use of the WT (60% of the use), followed by rehabilitation (40%). In the paper of Tranquille et al. [1], the WT was used particularly to train dressage horses (32%), eventers (16%) and jumpers (8%). The most important positive changes highlighted by the owners after the use of the WT were enhanced performance (77%) and improved strength and muscle development (38%). However, the authors described as a limitation of these data that the owners/riders were not asked about how they had perceived these improvements and therefore, it could have been a subjective appreciation. 

Several previous studies that analyzed the adaptations to exercise on a WT have suggested some possible benefits of this type of exercise for horses. WT exercise allows the horse to be exercised in a straight line (if there are two handlers, one on each side), on a firm surface, with a controlled velocity and with the potential of modifying water depth. Exercise in deep water, because of the buoyancy, reduces the load supported by the limbs. A three-to five-fold decrease in vertical ground reaction forces (vGRF) has been reported in human beings walking immersed in 1.3 m of water compared with results obtained while not immersed in water [2]. In dogs, Levine et al. [3] found that vGRF decreased proportionally with increasing water depth. Mean vGRF decreased by 9% after immersion to the level of the tarsal joints, by 15% after immersion at the level of the stifle joints and by 62% after immersion to the level of the hip joints in dogs [3]. Up to now, the reduction in vGRF with buoyancy has not been measured in horses, although McClintock et al. [4] made some calculations considering the Archimedes’ principle. It was found that horses with the water at the level of the tuber coxae experience a reduction around 75% of their body weight, whereas a reduction around 10-15% of body weight was described with the water at the level of the elbow [4]. However, these values were extrapolated from mathematical formulae and calculated in a tank filled with saline solution. The benefit associated with buoyancy is due to the reduction of the weight-bearing stress placed on joints and surrounding soft tissue structures [5,6]. Repetitive loading of the limb has been associated with detrimental bone and joint changes, including osteoarthritis. Therefore, reducing the impact of the limb during the stance phase of the stride as a consequence of the water buoyancy could be a useful strategy to extend the sport career of a horse by delaying the onset of these injuries. In fact, Greco-Otto et al. [7], using accelerometers that measured peak segmental limb acceleration and shock attenuation, demonstrated that WT exercise at the height of the stifle effectively reduces acceleration and increases shock attenuation through the forelimb. 

Other possible benefits of the exercise on a WT are associated with improvements in postural stability and limb functionality. In horses with a surgically induced osteoarthritis in the middle carpal joint, King et al. [8] found significant improvement in postural stability when the horses were exercised on a WT with the water at the level of the shoulder (supposed reduction of body weight: 60%). However, in the group of horses that were exercised on a land treadmill, this improvement was of minor intensity. Additionally, exercise on a WT resulted in a reduced synovial membrane inflammation together with improved thoracic limb loading and carpal joint flexion [9].

According to the data of Tranquille et al. [1], horse owners perceived an enhancement of strength and muscle development after WT training. Méndez-Angulo et al. [10] found increased flexion of the distal joints of the limbs in horses exercised at different water depths compared to horses exercised on a dry treadmill and because of this result, we measured the distribution of the total power (TP) into the three body axes, particularly to the dorsoventral axis (dorsoventral power, DVP). We also measured stride symmetry (SYM) and regularity (REG) in horses during WT exercises at different water depths. We aim to assess whether TP changes as a result of changes in the velocity and water depth and its consequences into the three body axes.

Three main hypotheses were proposed: first, due to the greater viscosity of the water compared to air, the horses would need to make a greater effort to move forward, and therefore, TP would increase with the depth of the water; second, this increase in TP would lead to a redistribution into the three body axes, with a more marked increase in DVP and dorsoventral displacement (DVD), because of the greater flexion of the distal joints of the limbs [10]; and third, as a direct consequence of the results shown by King et al. [8,9] regarding gait stability, we hypothesized that exercise on a WT at deeper depths would improve stride SYM and REG.

## 2. Material and Methods

### 2.1. Horses

Two trials were performed using two different groups of horses. Trial A was performed with six adult horses, of both sexes (two mares and four geldings) and of different breeds (four crossbred and two Andalusian horses) and trial B was carried out with five adult horses (two mares and three geldings), three crossbreds and two Andalusians. Body weight ranged between 390 and 460 kg (mean: 414 ± 45 kg), without significant differences between horses of trial A and B. 

Before starting the study, physical exam, hematology and clinical biochemistry analysis and lameness evaluation were performed in all horses. The lameness exam consisted of visual evaluation and lameness grade scoring according to the AAEP (American Association of Equine Practitioners). Flexion tests were also performed. Only sound horses were included in the study.

The horses of both trials were fully acclimatized to the WT exercise, because they were used for researching and teaching equine exercise physiology and sport medicine at the Veterinary School. Their fitness level was moderate and similar between horses within each trial, according to the results of a previous treadmill exercise test, measuring heart rate and blood lactate concentrations (data not presented). However, horses of trial B had lower fitness level than horses of trial A. 

### 2.2. Exercise on the WT

Horses of both trials were studied on the following situations, using a randomized design: with the WT without water (DT, dry treadmill; considered as baseline data), and with the water at the level of the fetlock (FET), carpus (CAR) and stifle (STF). The duration of each exercise session was of 40 min, including the time needed for filling and discharging the tank. In the case of the DT experiment, horses were exercised for 40 min on the WT without water. In all cases, the horses performed the exercise at walk. 

Exercise in trial A was performed at a velocity of 6.0 km/h, at the water depths of DT, FET and CAR. However, with the water at the STF level, the horses were not able to perform the exercise session at 6 km/h, since they were going backwards on the WT. For this reason, the velocity had to be reduced to 5 km/h. Because the changes in the accelerometric parameters in trial A could be attributable to both depth of the water, and the different velocities (6 km/h for DT, FET and CAR; 5 km/h for STF), a second trial was performed (trial B). In this second trial, horses were exercised at a velocity of 5.0 km/h in all the water levels (DT, FET, CAR, and STF).

### 2.3. Accelerometric Device

Horses wore a portable 3D gait analyzer (Equimetrix, Centaure-Metrix^®^), which integrates three orthogonal accelerometers that allow measuring accelerations along the three body axes, a data logger and a software program (Equimetrix-Centaure 3D^®^) to process acceleration signals. Positive values were obtained when accelerations were in dorsal, cranial and left directions. This accelerometer records data continuously while the horse is moving, at a sampling rate of 100 Hz. Additional information regarding this device has been published elsewhere [11,12,13].

In trial A the accelerometer was placed in two different locations, in the pectoral region (PECT) and over the midline of the sacrum region (SML). In the PECT location, the accelerometer was placed with a girth. In this position, the device is near the body’s center of gravity and with a good stability against the body of the horse. It has been indicated that in this location, the device provides more information regarding the acceleration patterns of the forelimbs. The accelerometer was also fixed in SML position with Velcro and an adhesive tape in order to obtain more information concerning acceleration patterns of the hindlimbs [12]. In the horses of trial B, the accelerometer was fixed only over the SML. In all cases, the same researcher (A.S.) fixed the device. 

### 2.4. Accelerometric Parameters

Stride energetic, coordination and spatiotemporal parameters were measured with the accelerometer in both positions (PECT and SML) in trial A (named trial A-PECT and trial A-SML) and in SML in trial B (named trial B-SML). 

Stride energetic parameters include dorsoventral power (DVP, W/kg), longitudinal power (LP, W/kg) and mediolateral power (MLP, W/kg). DVP is the integral of the power spectrum obtained by Fast Fourier Transformation (FFT) from the dorsoventral acceleration signal and measures limb suspension and loading activity. LP measures the craniocaudal or longitudinal activity and it is the integral of the power spectrum obtained by FFT from the longitudinal acceleration signal. LP measures the amount of acceleration and deceleration along the longitudinal axis. MLP is the side-to-side activity, calculated as the integral of the power spectrum obtained by FFT from the lateral acceleration signal. MLP therefore, measures the amount of acceleration and deceleration along the lateral axis. The sum of the three powers (DVP, LP and MLP) represents the total power (TP, W/kg). DVP, LP and MLP were expressed in absolute values and as percentages of TP in order to assess whether a redistribution of TP appears. Additionally, dorsoventral displacement (DVD) was calculated as an estimation from the double integration of the dorsoventral acceleration signal. 

Stride coordination parameters included regularity, REG (dimensionless) and symmetry SYM (dimensionless). REG measures the acceleration pattern similarity of successive strides in a period of time and SYM measures the similarities between left and right acceleration patterns [11,12,13]. 

Stride spatiotemporal parameters included stride frequency (SF, strides/s or Hz) and length (SL, m), provided that the velocity was fixed in the WT. 

For each horse, accelerometric parameters were calculated at each water depth (DT, FET, CAR, and STF) (twelve times; four measurements after 5 min of exercise with the tank filled at each depth; four measurements between 10 and 15 min of exercise; four measurements between 25 and 30 min of exercise), and with the accelerometric device in the two positions (PECT and SML) in the horses of trial A and in SML position in the horses of trial B. The data are presented as the means of these twelve measurements. It is unknown how long it takes to stabilize the locomotor pattern during an exercise on a WT. In land treadmill, when the horses are not acclimatized, it has been demonstrated that the locomotor pattern becomes stable after 5 min of exercise. After adaptation to the treadmill, experienced horses take at least 1 min for their gate to stabilize each time the treadmill starts moving or changed of velocity [14]. In the present research, we have been more conservative, and we did not perform any accelerometric measurement during the first 5 min of exercise after reaching the desired water depth.

When the accelerometer was fixed at PECT (trial A-PECT), horses were exercised under three conditions: DT, FET and CAR, but not with the water at the level of the SFT, since in this case, the device would have been submerged in water. 

### 2.5. Statistics

All data analyses were conducted using the statistical software Statistica for Windows (version 13.0 for Windows, USA). Normality of the data was evaluated with a Shapiro-Wilk’s W test. Because data were not adjusted to a normal distribution, non-parameters tests were used. Comparisons of the accelerometric variables for each trial and location of the accelerometer device were made between the different water depths with a Friedman repeated measures analysis of variance on ranks. When significance was found among the water depths for each accelerometric location and horse group, a Wilcoxon rank test was performed. The differences in percentages of the power in the three body axes were assessed with a Chi-square test.

Data are presented as means and standard deviations. Values of *p* < 0.05 were considered significant.

## 3. Results

**Figure 1:**
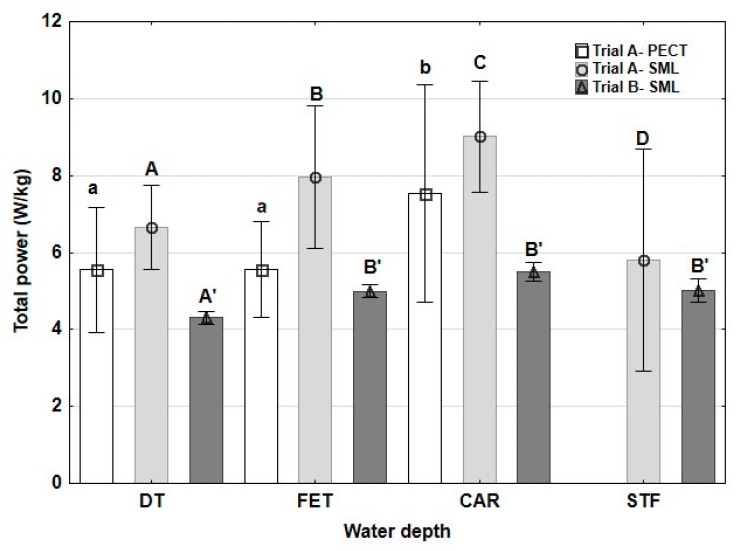
Means and standard deviations (whiskers) of total power measured in horses exercised on a water treadmill at different water depths and velocities. DT: dry treadmill, without water; FET: water at the level of the fetlock; CAR: water at the level of the CAR; STF: water at the level of the stifle. Trial A-PECT: accelerometer fixed in the pectoral region with velocities of 6 km/h; Trial A-SML: accelerometer fixed in the sacrum midline at velocities of 6 km/h with the water at DT, FET and CAR and at a velocity of 5 km/h with the water at STF; Trial B-SML: accelerometer fixed in the sacrum midline at a velocity of 5 km/h in the four water depths. Lowercase letters: significant differences between water depths in trial A-PECT; capital letters: significant differences between water depths in trial A-SML; capital letters with apostrophes: significant differences between water depths in trial B-SML. Different letters indicate significant differences between water depths at each trial *p* < 0.05.

Figure 1 shows TP values. TP was highest at the CAR depth in trial A- PECT. A progressive increase in TP was observed in trial A-SML, up to CAR depth, showing a significant decrease at STF when the velocity was reduced. In trial B-SML, and compared to DT, TP was significantly greater in the three conditions with water (FET, CAR and STF), without significant differences among the three of them. 

**Figure 2:**
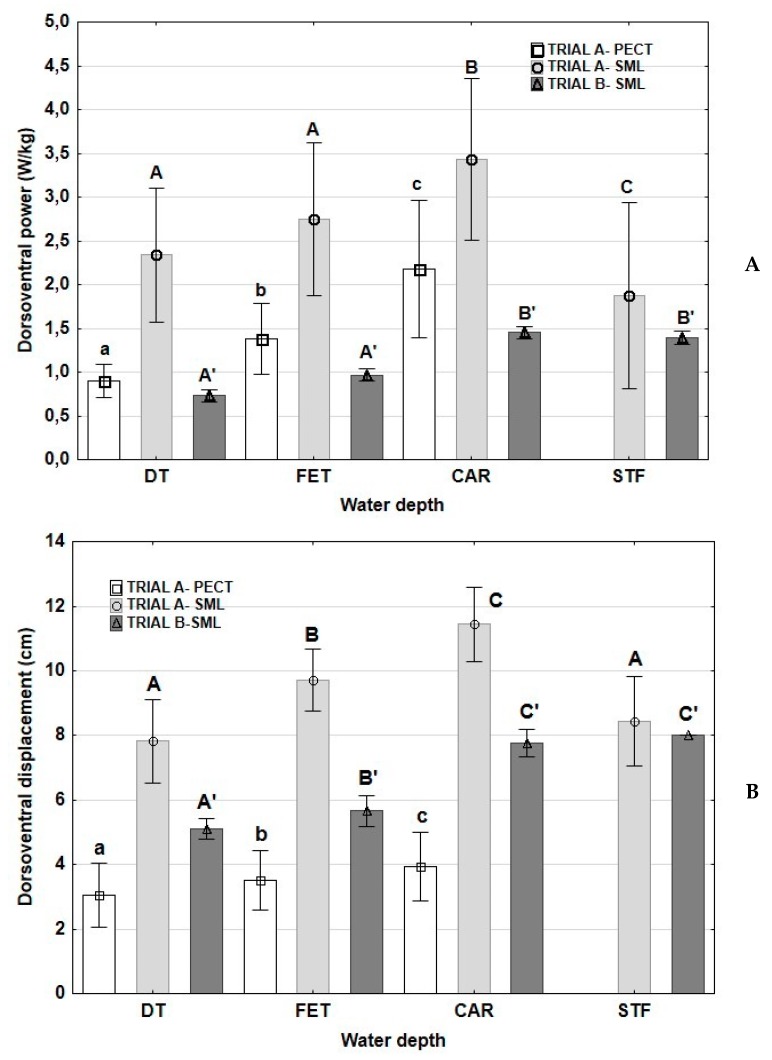
Means and standard deviations (whiskers) of dorsoventral power (**A**) and dorsoventral displacement (**B**) in horses exercised on a water treadmill at different water depths and velocities. DT: dry treadmill, without water; FET: water at the level of the fetlock; CAR: water at the level of the CAR; STF: water at the level of the stifle. Trial A-PECT: accelerometer fixed in the pectoral region with velocities of 6 km/h; Trial A-SML: accelerometer fixed in the sacrum midline at velocities of 6 km/h with the water at DT, FET and CAR and at a velocity of 5 km/h with the water at STF; Trial B-SML: accelerometer fixed in the sacrum midline at a velocity of 5 km/h in the four water depths. Lowercase letters: significant differences between water depths in trial A-PECT; capital letters: significant differences between water depths in trial A-SML; capital letters with apostrophes: significant differences between water depths in trial B-SML. Different letters indicate significant differences between water depths at each trial *p* < 0.05.

Values of DVP and DVD are depicted in Figure 2. In trial A-PECT, a progressive increase from DT-FET-CAR was found for both DVP and DVD. In trial A-SML, DVD increased from DT with the water depths of FET and CAR, decreasing at a water depth of STF. In the same trial, DVP increased from DT and FET with the water depth at CAR, decreasing at STF depth. In trial B-SML, DVD was significantly lower at DT compared to other water depths, without significant differences between CAR and STF. Also, in trial B-SML, DVP was statistically similar at DT and FET, increasing at CAR and STF, without significant differences between these last depths (Figure 2). 

**Figure 3:**
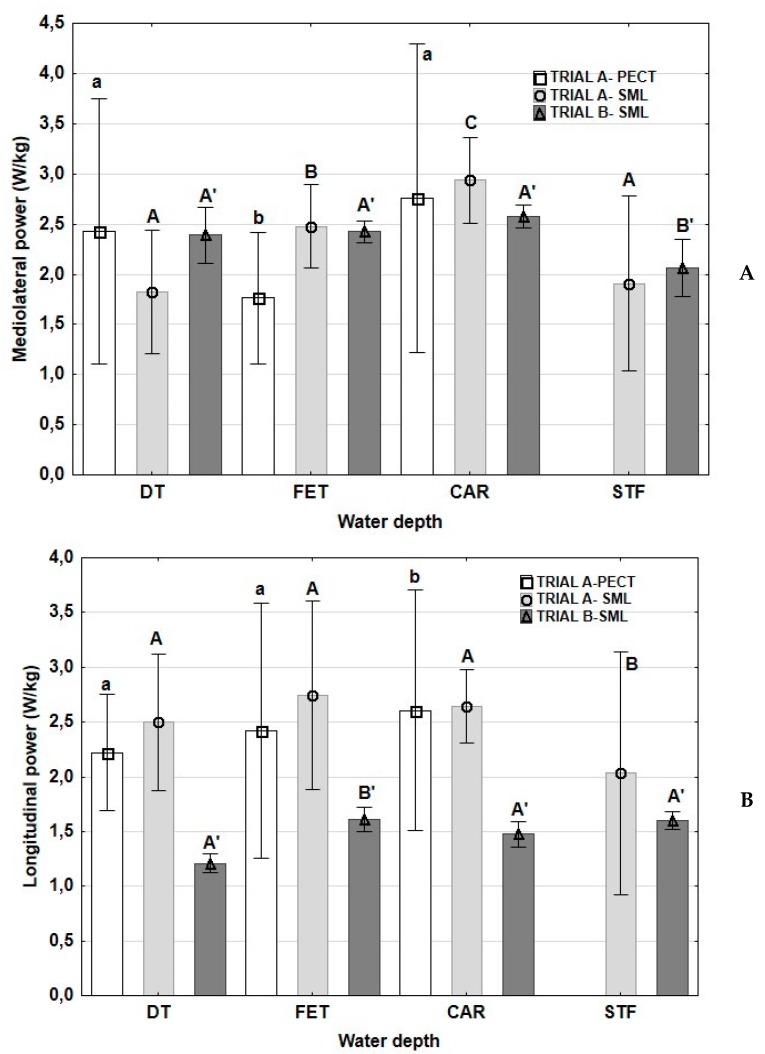
Means and standard deviations (whiskers) of longitudinal (**A**) and mediolateral (**B**) power in horses exercised on a water treadmill at different water depths and velocities. DT: dry treadmill, without water; FET: water at the level of the fetlock; CAR: water at the level of the CAR; STF: water at the level of the stifle. Trial A-PECT: accelerometer fixed in the pectoral region with velocities of 6 km/h; Trial A-SML: accelerometer fixed in the sacrum midline at velocities of 6 km/h with the water at DT, FET and CAR and at a velocity of 5 km/h with the water at STF; Trial B-SML: accelerometer fixed in the sacrum midline at a velocity of 5 km/h in the four water depths. Lowercase letters: significant differences between water depths in trial A-PECT; capital letters: significant differences between water depths in trial A-SML; capital letters with apostrophes: significant differences between water depths in trial B-SML. Different letters indicate significant differences between water depths at each trial *p* < 0.05.

Values of LP and MLP are shown in Figure 3. LP reached the greatest values with the water at the level of FET in trial A-PECT. This parameter did not change in trial A-SML at the water levels of DT, FET and CAR, and showed a significant decrease at STF depth. In trial B-SML, LP decreased at FET depth. MLP decreased at FET depth in trial A-PECT. A progressive increase in MLP was found in trial A-SML from DT to CAR, decreasing at STF, with values statistically similar to those found with the water at DT. In trial B-SML, MLP power did not vary from DT at FET and CAR but decreased at STF despite of the same velocity (Figure 3).

Table 1 shows the percentage of the three powers (DVP, LP and MLP) expressed as percentages of the TP. A redistribution of TP was found compared to DT, consisting of increases in DVP% at the expense of LP% and MLP% (Table 1). Stride SYM and REG values were not significantly modified by either water depth or velocity (Table 2). 

**Figure 4:**
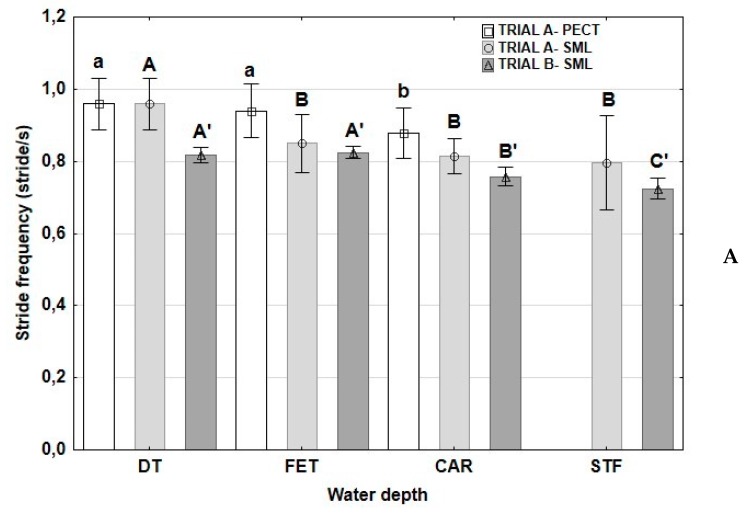

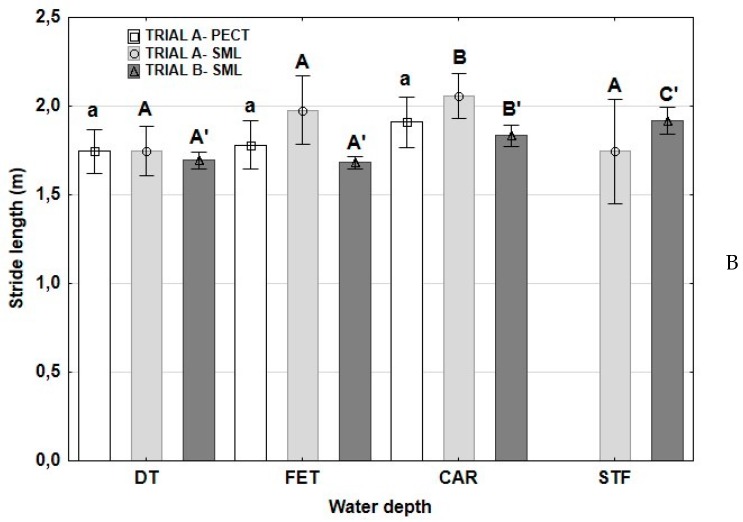
Means and standard deviations (whiskers) of stride frequency (**A**) and length (**B**) in horses exercised on a water treadmill at different water depths and velocities. DT: dry treadmill, without water; FET: water at the level of the fetlock; CAR: water at the level of the CAR; STF: water at the level of the stifle. Trial A-PECT: accelerometer fixed in the pectoral region with velocities of 6 km/h; Trial A-SML: accelerometer fixed in the sacrum midline at velocities of 6 km/h with the water at DT, FET and CAR and at a velocity of 5 km/h with the water at STF; Trial B-SML: accelerometer fixed in the sacrum midline at a velocity of 5 km/h in the four water depths. Lowercase letters: significant differences between water depths in trial A-PECT; capital letters: significant differences between water depths in trial A-SML; capital letters with apostrophes: significant differences between water depths in trial B-SML. Different letters indicate significant differences between water depths at each trial *p* < 0.05.

Values of SF and SL are presented in Figure 4. As expected, SF decreased and, consequently, SL increased with water depth in the three trials. When the velocity was reduced in trial A-SML, SL decreased, achieving values significantly similar to those found at DT. However, when the velocity was the same at all water depths, in trial B-SML, SL increased at CAR and further at STF water depth (Figure 4).

## 4. Discussion

The current research was designed in order to check three hypotheses. The first hypothesis was that exercise at the deepest water level studied (STF) would result in greater increases in TP compared with the lower water depths (i.e., DT, FET and, CAR). With the accelerometer in PECT location, the PT was significantly greater at CAR compared with DT and FET. Similarly, with the accelerometer at SML position, the PT was greater at the water depth of CAR, followed by FET and DT depths, findings that appears to confirm our first hypothesis. However, horses were not able to maintain the same velocity at the water level of the STF. Because of this, exercises at DT, FET and CAR were performed at 6 km/h, whereas those at STF depth were carried out at 5 km/h, which was a more comfortable velocity for the animals. This result could be attributed to the effect of the drag forces derived from the viscosity of the water, because the faster the horse moves, the greater the drag force is. When the velocity was reduced at the water depth of STF, a reduction in TP was found, reaching values even significantly lower than those found in DT conditions, which was an unexpected finding.

It has been demonstrated that power is dependent on velocity [11] and therefore, a decrease in velocity triggered a reduction in power. In addition to this, the greater water depth could also limit the advancement in the direction of the movement because of the direct effect of the buoyancy. To assess which of these factors (velocity and/or buoyancy) could have led to our results, we performed trial B, in which the horses were exercised at the same speed at all water depths. Unfortunately, because of logistic reasons, the same horses could not be used for both trials. Despite of the same velocity at all water depths (5 km/h), TP experienced a progressive increase from DT to FET and CAR depths, but it did not increase further with the water at the level of the STF, suggesting an important effect of the buoyancy at this water depth. Our results, consequently, suggest that increasing water depth from CAR to STF does not result in greater TP, contrary to what was firstly expected.

A reduction in TP has been described after administration of different sedatives such as xylazine, detomidine and romifidine [15] and acepromazine [16]. This reduction was attributed to the effect of the myorelaxation. Consequently, the increase in TP associated with water depth, from DT to CAR, observed in our research might be linked to a greater muscle strength. Muscle strength results from a combination of structural, neuromuscular, hormonal and metabolic factors, the first two being the most relevant. The structural factors associated with muscle strength depend on muscle histophysiological properties, such as cross-sectional area, fiber muscle typology, myofibrillar density and certain biomechanical factors, including the angle of insertion of muscle fibers in tendons, thickness, length and orientation of the tendons and the points of application of the strength in relation to the centers of joint rotation and the direction of the resulting forces [17,18]. It is known that these factors are modified by chronic exercise or training, but it is not likely that they might vary significantly in response to acute exercise (an exercise session), such as that performed in the current investigation. However, neuromuscular factors, that is the activation and recruitment processes of motor units, might vary during an exercise session. Therefore, it could be speculated that the variation in power with water depth in our study, not associated with changes in velocity, could have been determined by these neuromuscular factors. The pattern of activation of different agonist and antagonist muscles during water exercise at different velocities in the horse has been poorly studied. The most recent study on this subject study on this subject was carried out by King et al. [9], who found that horses with osteoarthritis in the middle carpal joint, induced by the creation of an osteochondral fragment with arthroscopy and subsequent land treadmill exercise, presented a delay in activation of various muscles of the distal aspect of the forelimb. After 8 weeks of exercise on a WT, with the water depth maintained at the point of the shoulder, the horses underwent a modification in the activation pattern of the deep digital flexor muscle, consisting of a reduction in the motor recruitment during the stance and swing phases of the stride, suggesting that recruitment of motor units could be modified with exercise in water in the horse.

In the study performed by Méndez-Angulo et al. [10], the amount of flexion and extension of the joints of the distal aspects of the fore- and hindlimbs of healthy horses exercised at different water depth was evaluated. An increase in the range of motion of the carpal, tarsal, metacarpophalangeal and metatarsophalangeal joints with exercise on the WT compared to baseline conditions (DT), primarily as a consequence of the increase in joint flexion was reported. These previous results, together with the expected increase in TP, led us to raise the second hypothesis of our investigation. Our second hypothesis was that as water depth increases, DVD will also increase. Consequently, in order to reach this greater DVD, DVP would increase, and also a greater percentage of the TP would be directed into this dorsoventral direction. This hypothesis was partially confirmed, because DVD and DVP in absolute values increased progressively from DT-FET-CAR, with the accelerometer in both positions. As happened with TP, with the accelerometer fixed at SML, significant lower values of DVD and DVP were observed with the water at the level of the STF when the velocity was reduced (trial A-SML), but not when the velocity remained constant at all depths (trial B-SML). These results reflect that, DVD and the power derived from this movement (DVP) depends not only on the depth of the water, but also on the velocity of movement.

Dressage horses are trained to improve their coordination, suppleness and gait collection, which means that the forward movement becomes more upward at the same time that SF slows down. Barrey et al. [19] reported that dressage horses experienced an increase in DVD with age and training, in correspondence with an increase in muscle power and collection, and significant differences exist between breeds which might explain why certain equine breeds have a locomotor profile more adapted to perform dressage. In addition, Biau and Barrey [20] found that young dressage horses with greater DVD at walk and trot and greater LP at the three gaits (walk, trot, and canter) obtained better scores in performance tests and also reached better performance during the first year of dressage competition. In jumping horses, the force produced for the hindlimbs for clearing the fences is one of the main factors affecting successful jump, because it determines the ballistic flight of the center of gravity, and therefore, greater values of DVD and DVP are advantageous for these horses [21]. In our research, we found that exercise on a WT led to significant increases in DVD and DVP with water depth (at to the level of CAR) compared to DT. If these modifications that occurred during the exercise on water were maintained during terrestrial locomotion, we will have scientific evidence to recommend this type of exercise for training dressage and jumping horses. However, until now, the changes in terrestrial locomotor after a training period on a water treadmill have not been evaluated. According to our results, this subject deserves to be investigated in the near future. 

The increase in DVD and DVP appeared to be a limiting factor to increase LP, which did not vary significantly with water depth and even was reduced. Similarly, when LP was expressed as a percentage of TP, a reduction was observed in the three trials, perhaps being a reflex of a redistribution of TP to the dorsoventral axis primarily. It is important to assess whether, after a WT exercise session, these modifications persist during terrestrial locomotion or conversely, if a greater TP results in different redistributions of TP into the three body axes depending on the exercise performed by the horse. If the first assumptions were fulfilled, the use of the WT to train horses that are very dependent on LP, such as endurance horses, would be a limitation. On the contrary, it would be very useful for dressage or jumping horses. However, if the second assumptions were fulfilled, any equine athlete would benefit from this modality of exercise, because the increased TP could be directed towards any of the three body axes depending on each type of performed exercise.

The third hypothesis of our study was that exercise on a WT would result in greater stability, as it has been shown in horses with induced carpal osteoarthritis after rehabilitation on a WT [8]. Consequently, greater values of stride SYM and REG were expected in our research. However, our data rejected this third hypothesis, since both SYM and REG have not been significantly influenced by velocity or water depth. The only difference in these parameters has been found in trial B, with the SYM values significantly higher during the exercise with the water at the FET depth compared to DT. However, this difference is of small magnitude and, in our opinion, it is too small to have practical relevance. The discrepancy between our data and those obtained by King et al. [8] might have some justifications. Firstly, King et al. [8] studied horses with induced lameness, which were rehabilitated for eight weeks, with daily exercise session on the WT, while in our case, the horses were sound, and they were subjected only to an exercise session on the WT. Secondly, the accelerometric measurements inside the tank probably were not the ideal procedure to determine the degree of stability. In fact, King et al. [8] used two serial force platforms to collect postural sway data under 3 stance conditions: normal square stance, base-narrow placement of the limbs and removal of visual cues.

In agreement with the results previously shown by other authors [10,22,23], deep water resulted in a reduction in SF and an increase in SL compared to shallow water, provided that the velocity is kept invariable. In our research, we have found that when the velocity was reduced at STF depth, SL was statistically similar to this found at DT and FET depths (Trial A-SML). However, when the velocity was the same in the four conditions (trial B-SML), SL increased, and SF decreased further from values obtained at CAR. These results derived from the combined effect of velocity and water depth have remarkable practical applications, since the synergy between both factors could be used to achieve specific locomotor objectives, such as increasing SL without or with increased TP, depending on the fitness level of each horse.

This article has several limitations. Only accelerometric changes during an exercise session at each water depth have been analyzed. It would be interesting to assess whether these adaptations to water depth are maintained during terrestrial locomotion, as well as to evaluate the effects during long-term training. In addition, this research has been carried out with a small number of animals and due to logistic reasons, the horses recruited for both trials were different. Horses of trial B were less fit than horses of trial A, and lower values for power was found. Consequently, no comparisons between trials were made, only between water depths within each trial. Both horses of trial A and B did not have special training, so it would be highly advisable to evaluate the effects of including exercise on a WT into a training program in order to achieve specific locomotor objectives, for example, to achieve greater DVP in dressage or jumping horses or to increase SL in the hindlimbs.

## 5. Conclusions

In conclusion, and despite these limitations, our research has confirmed that, total power increased with the depth of the water until reaching the CAR level. At SFT level, and supposedly, as a result of buoyancy, the power is not significantly greater than this found at CAR depth. According to these data, it could be hypothesized that horses could be exercised with the water at SFT level, increasing strength but with a substantial reduction in limb support, which would limit the risk of musculoskeletal injuries. In addition, velocity reduction leads to a decrease in TP, and therefore, the combination of variations in velocity and water depth can be very useful for training and particularly for rehabilitation, when the horses have reduced their fitness level. On the other hand, during a WT exercise session, DVD and DVP are increased, which would be useful for dressage and jumping horses training programs. Finally, the stride SYM and REG do not seem to be affected by water depth. 

## Figures and Tables

**Table 1:** Means and standard deviations of dorsoventral, longitudinal and mediolateral powers expressed as percentages of total power in horses exercised on a water treadmill at different water depths and velocities.

	DT	FET	CAR	STF
**Dorsoventral Power (% of Total Power)**				
**TRIAL A-PECT**	16.87 ± 3.43 ^a^	24.95 ± 5.90 ^b^	29.96 ± 8.06 ^c^	
**TRIAL A-SML**	34.38 ± 7.36 ^A^	34.12 ± 5.57 ^A^	37.52 ± 5.08 ^A^	31.84 ± 5.09 ^A^
**TRIAL B-SML**	17.09 ± 2.01 ^A’^	19.34 ± 1.33 ^A’^	26.48 ± 1.11 ^B’^	27.77 ± 1.61 ^B’^
**Longitudinal Power (% of Total Power)**				
**TRIAL A-PECT**	41.43 ± 9.31 ^a^	42.31 ± 12.60 ^a^	34.99 ± 7.64 ^b^	
**TRIAL A-SML**	37.14 ± 4.74 ^A^	34.00 ± 4.28 ^A^	29.70 ± 3.57 ^B^	35.01 ± 4.33 ^A^
**TRIAL B-SML**	28.18 ± 2.43 ^A’^	32.19 ± 1.36 ^B’^	25.83 ± 1.24 ^C’^	31.99 ± 1.86 ^B’^
**Mediolateral Power (% of Total Power)**				
**TRIAL A-PECT**	41.70 ± 10.03 ^a^	32.74 ± 12.93 ^b^	35.05 ± 10.22 ^b^	
**TRIAL A-SML**	28.48 ± 11.30 ^A^	31.88 ± 5.28 ^A^	32.78 ± 2.94 ^A^	33.15 ± 7.97 ^A^
**TRIAL B-SML**	54.63 ± 4.95 ^A’^	48.47 ± 1.54 ^B’^	47.69 ± 1.20 ^B’^	40.33 ± 3.53 ^C’^

DT: dry treadmill, without water; FET: water at the level of the fetlock; CAR: water at the level of the CAR; STF: water at the level of the stifle. Trial A-PECT: accelerometer fixed in the pectoral region with velocities of 6 km/h; Trial A-SML: accelerometer fixed in the sacrum midline at velocities of 6 km/h with the water at DT, FET and CAR and at a velocity of 5 km/h with the water at STF; Trial B-SML: accelerometer fixed in the sacrum midline at a velocity of 5 km/h in the four water depths. Lowercase letters: significant differences between water depths in trial A-PECT; capital letters: significant differences between water depths in trial A-SML; capital letters with apostrophes: significant differences between water depths in trial B. Different letters indicate significant differences between water depths at each trial *p* < 0.05.

**Table 2:** Means and standard deviation of stride symmetry and regularity in horses exercised on a water treadmill at different water depths and velocities.

	DT	FET	CAR	STF
**Stride Symmetry**				
**TRIAL A-PECT**	174.8 ± 43.08 ^a^	192.2 ± 53.64 ^a^	169.9 ± 43.62 ^a^	
**TRIAL A-SML**	258.2 ± 50.29 ^A^	225.9 ± 37.98 ^A^	237.3 ± 40.67 ^A^	213.6 ± 44.10 ^A^
**TRIAL B-SML**	204.1 ± 32.63 ^A’^	209.4 ± 49.04 ^A’^	213.9 ± 46.82 ^A’^	202.6 ± 46.21 ^A’^
**Stride Regularity**				
**TRIAL A-PECT**	191.9 ± 56.84 ^a^	182.9 ± 48.10 ^a^	192.7 ± 38.74 ^a^	
**TRIAL A-SML**	354.2 ± 39.07 ^A^	331.4 ± 38.32 ^A^	337.2 ± 27.31 ^A^	302.2 ± 37.30 ^A^
**TRIAL B-SML**	310.2 ± 38.91 ^A’^	304.7 ± 26.14 ^A’^	289 ± 32.28 ^A’^	321.7 ± 32.65 ^A’^

DT: dry treadmill, without water; FET: water at the level of the fetlock; CAR: water at the level of the CAR; STF: water at the level of the stifle. Trial A-PECT: accelerometer fixed in the pectoral region with velocities of 6 km/h; Trial A-SML: accelerometer fixed in the sacrum midline at velocities of 6 km/h with the water at DT, FET and CAR and at a velocity of 5 km/h with the water at STF; Trial B-SML: accelerometer fixed in the sacrum midline at a velocity of 5 km/h in the four water depths. Lowercase letters: significant differences between water depths in trial A-PECT; capital letters: significant differences between water depths in trial A-SML; capital letters with apostrophes: significant differences between water depths in trial B-SML. Different letters indicate significant differences between water depths at each trial *p* < 0.05.
